# Effect of chemical pretreatments on magnetic susceptibility of loess from Central Asia and the Chinese Loess Plateau

**DOI:** 10.1039/c8ra00617b

**Published:** 2018-03-20

**Authors:** Yougui Song, Yue Li, Qiansuo Wang, Hongmei Dong, Zhiping Zhang, Rustam Orozbaev

**Affiliations:** State Key Laboratory of Loess and Quaternary Geology, Institute of Earth Environment, Chinese Academy of Sciences Xi'an 710061 China syg@ieecas.cn; Department of Geosciences, Baylor University Waco TX 76798 USA Yougui_song@baylor.edu; College of Earth Science, University of Chinese Academy of Sciences Beijing 100049 China; College of Resources and Environment, Linyi University Linyi 276005 Shandong China; School of Management, Xi'an University of Science and Technology Xi'an 710054 China; Environmental Monitoring Station of Ili Kazakh Autonomous Prefecture Yining 835000 China; Institute of Geology, National Academy of Sciences of Kyrgyz Republic Bishkek 720040 Kyrgyzstan; Research Center for Ecology and Environmental of Central Asia (Bishkek) Bishkek 720040 Kyrgyzstan

## Abstract

Magnetic susceptibility (MS) as a paleoclimatic proxy plays an important role in paleoenvironmental reconstruction and past global climatic change. In order to discriminate the effect of composition on the MS of Quaternary eolian loess in inland arid Central Asia (CA), a series of comparative chemical experiments were designed to investigate the effects of different components on MS of loess from the Ili Basin CA and Chinese Loess Plateau (CLP). The results indicate that hydrochloric acid (HCl) can not only remove carbonate minerals, but also react with ferrous ions minerals by dissolving fine superparamagnetic particles (SPs), which reduces MS, especially in the CLP samples. Compared to the original samples, MS (*χ*_lf_) of acetic acid (AA) pretreated samples from CA and CLP increased by 20.3% and 4.8%, respectively, and their frequency-dependent MS (*χ*_fd_) increased by 53.4% and 13.0%, respectively, which indicates that the effect of carbonates on MS is greater for CA samples than for CLP samples. The variation in MS was below 5% in samples pretreated with perhydrol (H_2_O_2_) or distilled water, indicating that organic material and soluble components have very small influences on the MS. Temperature-dependence MS curves indicate that the transformation of magnetic minerals during the cooling of loess from the CLP mainly affected fine particles in the SPs, and that the contents of lepidocrocite and maghemite or goethite in the CA loess are lower than those in the CLP. The loess MS enhancement mechanism in Central Asia differs from that in the CLP.

## Abstract 2

Different chemical pretreat methods proved that the loess MS enhancement mechanism in Central Asia differs from that in the Chinese Loess Plateau (CLP).

## Graphical abstract

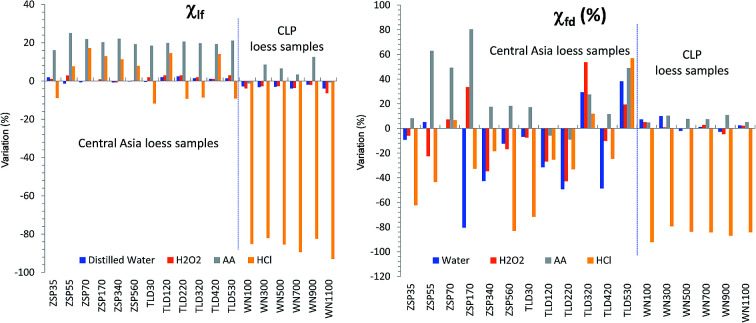

## 1. Introduction

In paleoclimatological studies of loess from the Chinese Loess Plateau (CLP)^1–4^ and European loess,^5–8^ magnetic susceptibility (MS) is regarded as an important climatological indicator and plays an important role in the study of past global change.^2,9–12^ The primary mechanisms that enhance the MS of Quaternary loess include pedogenesis,^13–15^ the dilution of falling cosmic dust,^16^ sediment compaction and carbonate leaching,^17^ and the decomposition of plant residues.^18^ The pedogenesis mechanism, which is widely accepted, suggests that the higher MS of paleosols relative to loess in the CLP is caused by the superparamagnetic particles (SPs) formed by pedogenesis.^13–15^ However, the mechanism causing MS enhancement in the loess of non-monsoon areas remains unclear (such as in the westerly wind zone in arid Central Asia).^19–26^ Lü *et al.*^27^ applied the citrate-bicarbonate-dithionite (CBD) method to study loess MS enhancement across the CLP and Tianshan in Central Asia. They found that the CBD method cannot provide pedogenetic information in the arid area of the Tianshan, and cannot distinguish between primary and secondary magnetic minerals.

The loess from arid CA often contains soluble salts, carbonates and organic materials, yet not studies have reported the influence of these compounds on MS. Carbonate minerals are anti-magnetic. Heller and Liu^17^ suggested that carbonates in loess reduce the MS, and that the leaching of carbonates can enhance the MS. Selective acid leaching experiments on loess indicate that hydrochloric acid (HCl) can dissolve carbonate materials and destroy many silicate minerals.^28^ Acetic acid (AA), however, can selectively leach carbonate components in loess and paleosols, but has a very small effect on silicates and iron oxides.^29^ There is a positive correlation between the organic mineral content and MS.^30,31^ Organic minerals have a distinct MS, which depends on the degree of carbon polymerization, and they can also vary from paramagnetic to diamagnetic.^32^ In order to determine the effect of composition on the MS of eolian loess in the inland arid CA, the authors conducted a series of comparative experiments to remove organic material/soluble salt using distilled water or perhydrol (H_2_O_2_), and furthermore remove carbonates using HCl or AA. The results were compared with those using loess samples from the CLP.

## 2. Setting and sampling

**Fig. 1:**
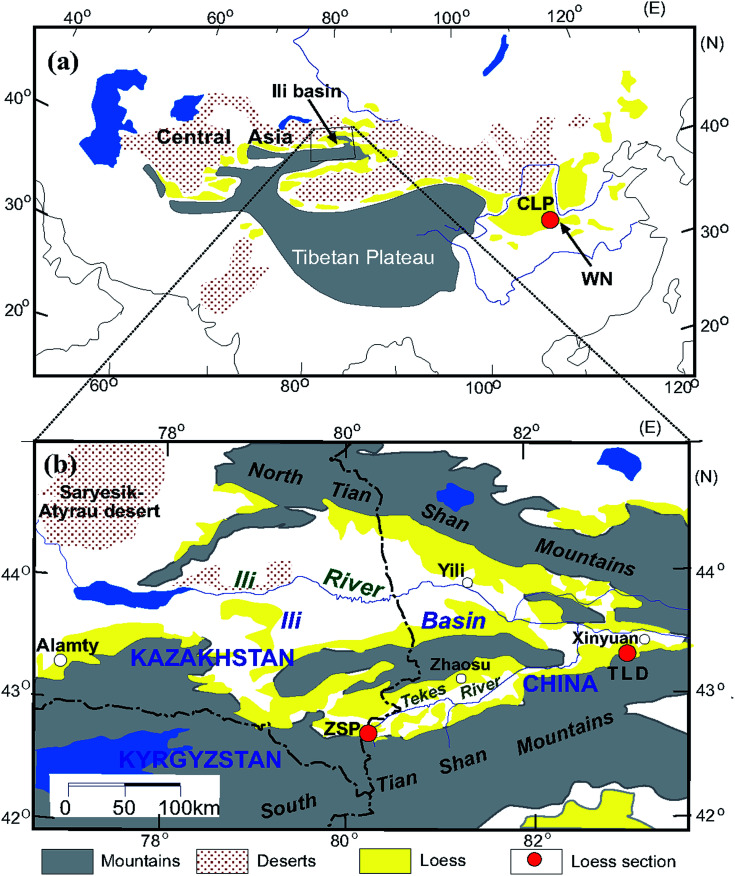
Loess sections locations in the Chinese Loess Plateau (a) and the Ili Basin of Central Asia (b) (modified from Song *et al.*, 2014).

The trumpet-shaped Ili Basin, surrounded by branches of the Tianshan Mountains in Central Asia, is a favourable geomorphological setting for dust deposition. Loess is widely distributed across the Ili Basin, mainly on different terraces of the Ili, Kunes, Tekes and Kashi rivers; the piedmont; and desert margins.^33^ The loess in the Ili Basin has typical characteristics of eolian deposits.^33^ In total, 12 loess and paleosol samples were collected from the Zhaosu Poma (ZSP) section (80.25°E, 42.69°N)^34–37^ and Talede (TLD) section (83.01°E, 43.42°N)^19,38,39^ at the southern margin of the Ili Basin (Fig. 1). Both loess sections are situated on river terraces, close to the southern Tianshan Mountains. The ZSP loess section is 6.9 m thick and has mainly been deposited since the last glacial period.^20^ The TLD loess section is approximately 30 m thick^39,40^ (Fig. 1b), and we collected loess samples from the uppermost 6 m. Because the MS enhancement mechanism of the CLP loess has been well documented during the past several decades,^3,11,15,17,41–43^ here we select 6 samples from the Weinan (WN) loess section (109.6°E, 34.5°N) deposited since the last interglacial period^44^ on the south-eastern margin of the CLP (Fig. 1a) for comparison. A comprehensive comparative study is helpful for understanding the differences and similarities of MS enhancement between these sites.

## 3. Methods

### 3.1 Pretreatment

**Fig. 2:**
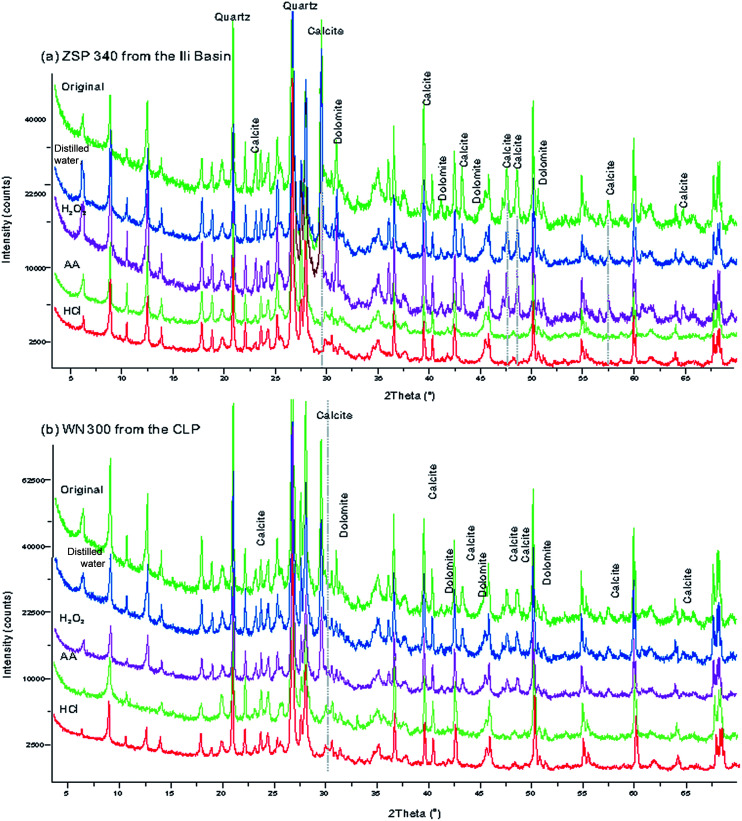
X-ray diffraction patterns with different pretreated methods of selected samples from the Ili Basin (a) and the CLP (b).

The loess samples were dried in an oven at a temperature of 50 °C, and were then thoroughly mixed by coarsely grinding in an agate mortar. For both the CA loess and the CLP loess, four 15 g subsamples of each sample were retrieved and marked as A, B, C and D. The subsamples were placed in a 450 ml centrifuge cup after measuring the MS. Next, 50 ml of distilled water was added to the A samples to remove soluble salt; 50 ml of H_2_O_2_ solution (10%) was added to the B subsamples to remove organic materials; 50 ml of AA (10%) was added to the C subsamples to remove calcium carbonate; 50 ml of HCl (10%) was added to the D subsamples to remove carbonate minerals and possibly other ferrous minerals. All subsamples were stirred multiple times and left to stand for more than 48 h to allow the subsamples to effectively disperse and fully react. Distilled water was added to the centrifuge cup, the subsamples were centrifuged at 5000 rpm for 15 min, and the clear water at the top of the cup was decanted. This process was repeated until the pH value of the water became neutral. Residues were collected and dried at a constant temperature of 50 °C. The subsamples were weighed, and the MS was measured. To test the degree of carbonate removal, we used an X-ray diffractometer to identify variations in mineralogy (Fig. 2). The air-dried loess sediments were ground by hand using an agate mortar and pestle to about 300 mesh size (<40 μm), then the powder samples were scanned from 3 to 70° (2*θ*) at generator settings of 40 mA and 40 kV using a Philips X'pert Pro (PW3071) X-ray diffractometer with 1.540598 Å CuKα radiation. The diffraction spectrum did not show the characteristic peaks of carbonate minerals such as calcite, dolomite, which indicates that they were removed completely (Fig. 2). After the subsamples were fully reacted with an H_2_O_2_ solution and dried at 50 °C, they were baked at 550 °C. The weights did not change significantly, which indicates that the organic minerals were thoroughly removed by this method.

### 3.2 Magnetic measurements

Magnetic susceptibility measurements were made on 10 g dry powder samples at low (LF = 0.47 kHz) and high (HF = 4.7 kHz) frequencies in a Bartington Instruments dual frequency MS2B sensor and expressed as mass-specific magnetic susceptibility (*χ*_lf_, *χ*_hf_), mass-specific frequency-dependent susceptibility (*χ*_fd_) and percentage frequency-dependent susceptibility (*χ*_fd_%). *χ*_fd_ is defined as *χ*_lf_ − *χ*_hf_, and *χ*_fd_% is defined as (*χ*_lf_ − *χ*_hf_)/*χ*_lf_ × 100%. The MS variation curve (χ–*T*) of MS with temperature was measured using an AGICO Inc KLY-3s Kapabridge and CS-3 temperature control system with approximately 0.2 g of powder samples. To prevent the samples from being oxidized during heating, the entire experiment was performed in an argon environment. All measurements were completed in the State Key Laboratory of Loess and Quaternary Geology, Chinese Academy of Sciences (CAS).

## 4. Results

**Fig. 3:**
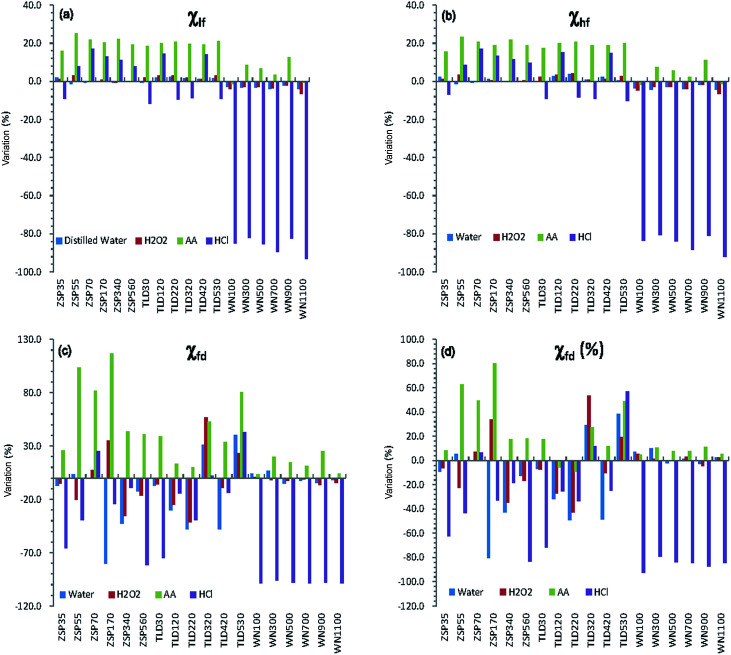
Column diagrams of magnetic parameters variations relative to the original samples from Central Asia (prefixed by ZSP and TLD) and the Chinese Loess Plateau (prefixed by WN) pretreated with different methods.

Table 1 shows the values of magnetic parameters (*χ*_lf_, *χ*_hf_, *χ*_fd_, *χ*_fd_%) of all samples before and after pretreatment with different methods. Generally, both mass-specific magnetic susceptibility (*χ*_lf_, *χ*_hf_) and frequency-dependent susceptibility (*χ*_fd_, *χ*_fd_%) in the original samples from the CA are significantly lower than those of loess samples from the CLP. In particular, the values of frequency-dependent susceptibility are very low (<5 × 10^−8^ m^3^ kg^−1^ or <5%) (Table 1). The absolute differences between CLP samples are larger than those of the CA samples. The maximum *χ*_lf_ difference in value of WN1100 from the CLP reached 247.2 × 10^−8^ m^3^ kg^−1^. The absolute *χ*_lf_ differences of the CA samples were clearly smaller, with a maximum of 13.0 × 10^−8^ m^3^ kg^−1^. However, most of the percentage increases or decreases in the CA loess were greater than those of the CLP loess, except HCl (Fig. 3). The most prominent changes in magnetic parameters were that all HCl-pretreated samples from the CLP decreased significantly, with average percentage decreases ranging from 84.9% to 98.0% (Fig. 3). In contrast, both *χ*_lf_ (or *χ*_hf_) and *χ*_fd_ (or *χ*_fd_%) of loess samples from CA showed no statistically significant changes after the samples were pretreated with HCl (Fig. 3 and Table 1). Magnetic parameters of most samples (except WN100 and WN1100) increased after they were pretreated with AA, and the increments of the CA samples were obviously higher than those of the CLP samples (Fig. 3). The mean values of *χ*_lf_ and *χ*_hf_ in the CA samples increased by 20.3% and 19.6%, respectively, but their increments in the CLP samples were only 4.8% and 3.9%, respectively (Table 1). Mean values of *χ*_lf_ and *χ*_hf_ in CA and CLP samples pretreated with distilled water and H_2_O_2_ varied by less than 4%, while *χ*_fd_ variations exceeded 17% in most CA samples pretreated with distilled water. *χ*_fd_ and *χ*_fd_% variations in both CA and CLP samples were less than 5% when pretreated with H_2_O_2_.

**Table 1:** Variations of magnetic parameters of samples used different chemical pretreatment methods

Pretreated method		*χ*_lf_ (10^−8^ m^3^ kg^−1^)					*χ*_hf_ (10^−8^ m^3^ kg^−1^)					*χ*_fd_ (10^−8^ m^3^ kg^−1^)					*χ*_fd_ (%)				
		Original	Water	H_2_O_2_	AA	HCl	Original	Water	H_2_O_2_	AA	HCl	Original	Water	H_2_O_2_	AA	HCl	Original	Water	H_2_O_2_	AA	HCl
Central Asia samples	ZSP35	78.9	80.4	79.8	91.6	71.8	76.3	78.0	77.3	88.3	71.0	2.6	2.4	2.5	3.3	0.9	3.3	3.0	3.1	3.6	1.2
	ZSP55	76.4	75.4	78.6	95.5	82.3	74.7	73.6	77.2	92.0	81.2	1.7	1.8	1.4	3.5	1.0	2.2	2.4	1.7	3.7	1.3
	ZSP70	72.4	71.9	72.4	88.2	84.9	71.1	70.6	71.0	86.0	83.3	1.2	1.2	1.3	2.3	1.6	1.7	1.7	1.9	2.6	1.8
	ZSP170	59.8	59.9	60.4	72.0	67.6	59.0	59.7	59.2	70.1	66.9	0.9	0.2	1.2	1.9	0.7	1.4	0.3	1.9	2.6	1.0
	ZSP340	43.1	42.8	42.8	52.7	48.0	42.3	42.3	42.3	51.5	47.3	0.8	0.4	0.5	1.1	0.7	1.8	1.0	1.2	2.1	1.5
	ZSP560	47.1	47.0	47.2	56.1	50.8	46.1	46.2	46.4	54.8	50.6	0.9	0.8	0.8	1.3	0.2	2.0	1.7	1.7	2.4	0.3
	TLD30	82.2	81.8	83.8	97.4	72.5	78.8	78.6	80.5	92.6	71.7	3.4	3.2	3.2	4.8	0.9	4.2	3.9	3.9	4.9	1.2
	TLD120	80.3	82.0	82.7	96.3	92.1	78.5	80.7	81.3	94.2	90.5	1.9	1.3	1.4	2.1	1.6	2.3	1.6	1.7	2.2	1.7
	TLD220	82.0	84.0	84.4	98.9	74.4	79.9	82.9	83.1	96.6	73.1	2.1	1.1	1.2	2.3	1.3	2.6	1.3	1.5	2.3	1.7
	TLD320	80.3	81.4	81.9	96.1	73.3	78.5	79.1	79.1	93.4	71.5	1.8	2.3	2.8	2.7	1.8	2.2	2.9	3.4	2.8	2.5
	TLD420	90.0	91.0	91.0	107.3	102.7	88.1	90.0	89.2	104.8	101.0	1.9	1.0	1.7	2.6	1.6	2.1	1.1	1.9	2.4	1.6
	TLD530	81.0	82.2	83.4	98.1	73.6	79.4	80.0	81.5	95.3	71.3	1.6	2.2	1.9	2.8	2.2	1.9	2.7	2.3	2.9	3.1
CLP samples	WN100	154.2	149.9	148.2	152.2	22.9	138.2	133.1	132.0	135.6	22.7	16.0	16.7	16.2	16.6	0.2	10.4	11.2	10.9	10.9	0.8
	WN300	122.8	118.9	119.4	133.3	22.1	111.4	106.7	108.2	119.6	21.6	11.4	12.1	11.2	13.7	0.4	9.3	10.2	9.4	10.3	1.9
	WN500	155.2	150.6	151.0	165.4	22.6	139.9	136.0	136.1	147.8	22.2	15.4	14.6	15.0	17.7	0.4	9.9	9.7	9.9	10.7	1.6
	WN700	220.2	211.5	212.1	227.6	23.4	196.7	188.7	188.8	201.5	23.0	23.5	22.8	23.3	26.1	0.4	10.7	10.8	11.0	11.5	1.7
	WN900	121.3	119.0	118.8	136.5	21.3	110.0	108.2	108.3	122.4	21.1	11.3	10.8	10.6	14.1	0.3	9.3	9.1	8.9	10.4	1.2
	WN1100	265.8	255.4	248.9	263.8	18.6	234.9	224.9	219.2	231.6	18.3	31.0	30.5	29.6	32.3	0.3	11.6	11.9	11.9	12.2	1.8
CA mean		72.8	73.3	74.0	87.5	74.5	71.1	71.8	72.3	85.0	73.3	1.7	1.5	1.7	2.6	1.2	2.3	2.0	2.2	2.9	1.6
CLP mean		173.3	167.5	166.4	179.8	21.8	155.2	149.6	148.8	159.7	21.5	18.1	17.9	17.6	20.1	0.3	10.2	10.5	10.3	11.0	1.5

## 5. Discussion

In samples pretreated with HCl, the *χ*_lf_ and *χ*_fd_ of CLP samples decreased from means of 173.4 × 10^−8^ to 21.8 × 10^−8^ m^3^ kg^−1^ and 18.1 × 10^−8^ to 0.3 × 10^−8^ m^3^ kg^−1^ (Table 1), respectively, indicating a greater than 86% reduction in *χ*_lf_ and 98% reduction in *χ*_fd_. Meanwhile, there were no significant changes in CA samples. The *χ*_fd_ is the difference between *χ*_lf_ and *χ*_hf,_ and is thus regarded as an indicator to reflect the formation of the fine magnetic particles near the threshold (∼20–25 nm)^45^ of the stable domain (SD) and superparamagnetic (SP) of ferromagnetic minerals during the continuous pedogenesis of soils. *χ*_fd_ can be used to measure the absolute concentration of SP and its contribution to MS.^10,46,47^ While *χ*_fd_% is controlled by both grain size distribution and the concentration of particles near the SP/SD threshold,^47^ it is sensitive only to a very narrow portion of the grain size distribution near the SP/SD threshold. Therefore, the MS enhancement of the CLP is mainly caused by SPs formed during pedogenesis,^11^ further supporting the pedogenetic model for the CLP. Here, *χ*_fd_% values less than 5% indicate that SPs have a limited contribution to the MS.^35,48^ Specifically, *χ*_fd_% values (<5%) of the CA loess samples indicate that the enhanced MS of the Ili loess is mainly associated with coarse particles.

In samples pretreated with AA, the *χ*_fd_ of loess samples from CA and the CLP increased by 0.9 × 10^−8^ and 2.1 × 10^−8^ m^3^ kg^−1^, respectively, amplifying initial values by 53.4% and 13.2%, respectively. Due to the effect of secondary carbonates on the MS of loess, their removal will cause relative enrichment of the magnetic minerals, thus increasing the MS. Compared to the CLP samples, the removal of carbonates increased the *χ*_lf_ of loess samples from CA by an average of 14.7 × 10^−8^ m^3^ kg^−1^. *χ*_lf_ increased by an average of 6.6 × 10^−8^ m^3^ kg^−1^ in loess samples from the CLP, which is much smaller than that for loess samples from CA. Because CA is an arid inland area, a larger amount of secondary carbonate is formed during soil formation,^49^ and there is a greater accumulation of carbonates than on the CLP. Except for the part of the paleosol that experienced eluviation, loess, weakly developed paleosol, and ancient soil are abundant in carbonates. Therefore, the influence of carbonates on MS is more significant in loess samples from CA than in those from the CLP.

The mean *χ*_fd_ of CA samples pretreated with distilled water was reduced by 11.8%, and the *χ*_lf_ of CA samples increased slightly (by 0.6%). Meanwhile, both *χ*_lf_ and *χ*_fd_ in the CLP samples showed no obvious changes. The variations in mean values of *χ*_lf_, *χ*_hf_, *χ*_fd_ and *χ*_fd_ from both CA and CLP samples pretreated with H_2_O_2_ and distilled water were less than 5%, except in the *χ*_fd_ and *χ*_fd_% of CA samples pretreated with distilled water (Fig. 3), which indicates that the soluble mineral content and organic minerals in the loess samples have a very small effect on the MS. This result is also supported by the consistency (almost a complete overlap) between the *χ*_lf_ curves for the original samples and those pretreated with distilled water or H_2_O_2_. The change in *χ*_fd_ and *χ*_fd_% was relatively large for CA samples, which is most likely due to the relatively small SPs in CA loess and paleosol (generally 0.8–2.5 × 10^−8^ m^3^ kg^−1^) samples. The background values measured in air after finishing measurement of samples were generally (−0.3 to 0.3) × 10^−8^ m^3^ kg^−1^, which caused a relatively large variation. Therefore, the change in *χ*_fd_ does not properly reflect the change in the SPs content.

**Fig. 4:**
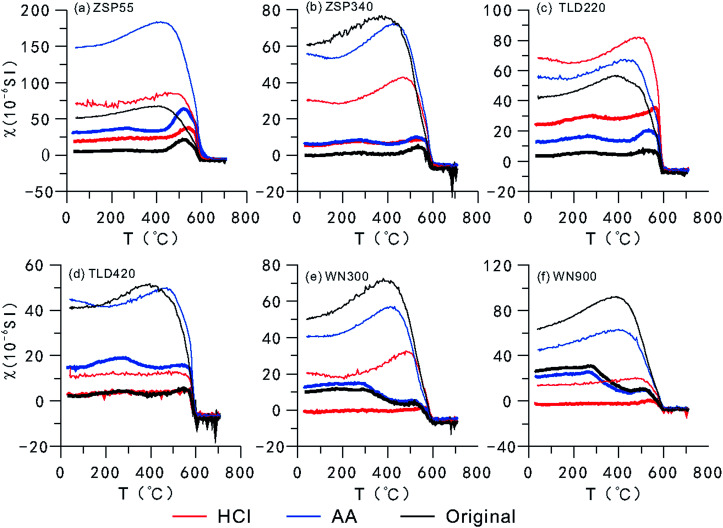
The MS-temperature curves for loess samples pretreated with different methods from Central Asia and the Chinese Loess Plateau. Bold and thin lines represent heating and cooling, respectively, and red and blue lines represent samples pretreated with HCl and AA, respectively.

A curve showing the variation in MS with temperature (TDS curve) (Fig. 4) demonstrates that the magnetic minerals transform during the thermal demagnetization process.^50–54^ The cooling curve is superposed on the heating curve, which indicates that new magnetic minerals are formed during the cooling process.^20,46,50,52,54^ As shown in Fig. 4, the MS of loess from the two areas abruptly decreases or increases during heating or cooling to near 580 °C, which indicates that magnetite is the main contributor to MS and is the dominant component of the newly-formed magnetic minerals. The number of magnetic minerals formed during cooling can be determined based on how much higher the cooling curve is relative to the heating curve. According to the results of CLP samples (Fig. 4e and f), there were few new magnetic minerals formed in the HCl-pretreated samples during the cooling process, and there was no significant difference between the original samples and the AA-pretreated samples, which indicates that the fine particle fraction in SPs most likely had a relatively significant contribution to the transformation of magnetic minerals. Because the SP contents were relatively lower in the Ili loess, CA, and although their size was relatively large, the difference is not obvious except for in samples B and D. Meanwhile, during the heating process, the original samples and the AA-pretreated samples from the CLP exhibited a peak near 260 °C and 520 °C, respectively, most likely caused by the transformation of lepidocrocite to maghemite^55^ and the formation of magnetite from the pyrolysis of iron silicate minerals or clay minerals.^35^ Between 300 °C and 440 °C, the MS decreases as the temperature increases, which is generally attributed to the transformation of maghemite or goethite into hematite.^53–55^ In CA samples, these characteristics were indistinct in all samples except for D, which was pretreated with AA. This indicates that there is less lepidocrocite and maghemite or goethite in the Ili Basin loess than in the CLP loess. During the heating of the HCl-pretreated samples, the CA and CLP samples did not exhibit peaks near 260 °C, and decreased from 300 °C to 440 °C. Therefore, the numbers of lepidocrocite and maghemite or goethite particles in the SPs are most likely very limited.

## 6. Conclusions

A comparison of *χ*_lf_ and *χ*_fd_ for HCl-pretreated loess samples from CA and the CLP indicates that HCl dissolves the carbonates in the samples, and also reacts with the Fe ions of the magnetic minerals, decomposing the fine minerals in the SPs and causing the MS to decrease. The magnetic minerals in the loess from the Ili Basin, CA, mainly comprise coarse particles, and they have a relatively small contribution to the SP and the MS. *χ*_lf_ and *χ*_fd_ of AA-pretreatment CA samples were increased by 20.3% and 53.4%, respectively, while those of CLP samples only increased by 4.8% and 13.2%, respectively. AA affects MS to some extent, because carbonates have a much more significant influence on the MS of loess from CA than from the CLP. For most samples pretreated with H_2_O_2_ or distilled water, the variations in *χ*_lf_ were less than 5%, which indicates that the soluble mineral contents and organic materials in the loess have a very small effect on the MS. Comparative analysis of TDS curves indicates that the transformation of magnetic minerals during the cooling of loess from the CLP mainly affected fine particles in the SP, and that the contents of lepidocrocite and maghemite or goethite in the Ili loess were lower than those in the CLP. The experiments indicate that the process of loess MS enhancement in Central Asia differs from that on the CLP, consistent with other geochemical and magnetism investigations.^19–21,35,39,56,57^ These results help in understanding the paleoclimatic significance and paleoenvironmental reconstruction in the Central Asia arid area.

## Conflicts of interest

We have read your policy on conflict of interest and confirm that there are no conflicts to declare.

## Supplementary Material
